# Current insights into the safety and adverse effects of methylphenidate in children, adolescents, and adults - narrative review

**DOI:** 10.1007/s43440-025-00763-0

**Published:** 2025-07-22

**Authors:** Andrzej Silczuk, Aleksandra Lewandowska, Małgorzata Filip, Paweł A. Atroszko, Jakub Podolec, Małgorzata Gałecka, Robert Madejek, Łukasz Czyżewski

**Affiliations:** 1 Department of Community Psychiatry, Faculty of Life Sciences, Medical University of Warsaw, Warsaw, Poland; 2 Children Psychiatry Unit, Specialized Psychiatric Health Care Centre in Łódź, Łódź, Poland; 3 Department of Drug Addiction Pharmacology, Maj Institute of Pharmacology, Polish Academy of Sciences, Kraków, Poland; 4 Department of Psychometrics and Statistics, Institute of Psychology, Social Sciences Faculty, University of Gdańsk, Gdańsk, Poland; 5 Department of Interventional Cardiology, Institute of Cardiology, Jagiellonian University Medical College, The John Paul II Hospital, Kraków, Poland; 6 Department of Psychotherapy, Medical University of Łódź, Łódź, Poland; 7 Department of Geriatric Nursing, Faculty of Life Sciences, Medical University of Warsaw, Warsaw, Poland

**Keywords:** Methylphenidate, ADHD, Safety, Adverse effects

## Abstract

Methylphenidate (MPH) is a central nervous system stimulant that is approved and widely used for the treatment of attention-deficit hyperactivity disorder (ADHD) and narcolepsy. It acts primarily by inhibiting the reuptake of dopamine and norepinephrine, thereby enhancing synaptic concentrations of these neurotransmitters and improving attention, impulse control, and wakefulness. Despite its well-established therapeutic efficacy, MPH is associated with a complex safety profile that necessitates careful consideration, particularly in long-term use and in populations with preexisting health conditions. Cardiovascular risks, including increased heart rate, elevated blood pressure, and, in rare cases, serious adverse events such as myocardial infarction, arrhythmias, and sudden cardiac death, have been reported. Psychiatric adverse effects, including anxiety, agitation, psychotic symptoms, and exacerbation of preexisting mood disorders, also warrant close monitoring. Additionally, MPH has the potential for misuse, abuse, and dependence, particularly due to its dopaminergic effects, which can contribute to reinforcement and addiction-related behaviors. This review synthesizes current evidence on the safety of MPH, with a focus on its impact on cardiovascular and psychiatric health, and addiction potential. Special attention is given to vulnerable populations, including children, adolescents, individuals with comorbid psychiatric or cardiovascular conditions, and those with a history of substance use disorders. Furthermore, sex and gender influence health outcomes, for MPH healthcare strategies have been addressed. Given these concerns, the necessity for rigorous patient monitoring, individualized risk assessment, and adherence to prescribing guidelines is emphasized to optimize therapeutic outcomes while minimizing risks.

Clinical trial number: Not applicable.

## Introduction

Methylphenidate (MPH) remains a first-line pharmacological intervention for attention-deficit hyperactivity disorder (ADHD) across pediatric and adult populations, as well as for narcolepsy [1–3]. Current clinical guidelines emphasize that cognitive-behavioral therapy (CBT) may be recommended as the first-line treatment for ADHD, particularly in pediatric populations [4]. When pharmacological intervention is warranted, such as in moderate to severe cases, MPH and other stimulant medications are considered the drugs of choice [4]. The clinical efficacy of MPH in ADHD is primarily attributed to its capacity to inhibit the human dopamine transporter (hDAT), thereby increasing extracellular dopamine concentrations in critical brain regions such as the prefrontal cortex and striatum, which underpin attention and executive functions [5, 6].

Recent high-resolution cryo-electron microscopy (cryo-EM) studies have elucidated the molecular interactions between MPH and hDAT, revealing that MPH binds to the orthosteric site of the transporter and stabilizes it in an outward-facing conformation that impedes dopamine reuptake [5]. Specifically, the phenyl and piperidinyl groups of MPH interact with key amino acid residues - F76, D79, F326, and S422 - which are critical for high-affinity ligand binding. Mutagenesis experiments have confirmed the indispensable roles of residues F326 and S422 in MPH recognition [5]. This binding mode sterically obstructs conformational transitions of transmembrane domains TM1b and TM6a, necessary for substrate translocation, thus effectively inhibiting dopamine uptake. Notably, MPH’s binding mechanism contrasts with that of other DAT inhibitors such as GBR12909 or benztropine, which stabilize inward-facing conformations and are associated with lower abuse potential, suggesting that MPH’s unique interaction pattern may contribute to its reinforcing properties and abuse liability [5].

Pharmacokinetically, MPH is available in both immediate-release (IR) and extended-release (ER) formulations. IR preparations are characterized by rapid absorption, resulting in peak plasma concentrations typically reaching approximately 2 h post-oral administration, with an effect duration ranging from 1 to 4 h and an elimination half-life (t½) of about 2 to 3 h [3, 6]. In contrast, ER formulations, such as the oral suspension NWP06, provide a more gradual absorption profile that sustains dopaminergic stimulation over an extended period, thereby optimizing clinical management by reducing dosing frequency and attenuating peak-related adverse effects. Specifically, the maximum plasma concentration (Cmax) for NWP06 is approximately 13.61 ng/mL, compared to 20.94 ng/mL for IR MPH. The time to reach peak concentration (Tmax) is around 5 h for NWP06, while it is approximately 7.33 h for IR MPH. Additionally, the terminal elimination half-life (t½) of NWP06 is longer, at about 5.65 h, relative to 3.74 h observed for the IR formulation [3, 7, 8].

Despite its primary dopaminergic action, in vitro data have indicated MPH interaction with serotonergic receptors, including 5-HT2B and 5-HT1A subtypes, though the functional consequences of these interactions remain to be fully elucidated [9, 10]. Moreover, neurochemical studies have documented MPH-induced modulation of glutamatergic transmission in the anterior cingulate cortex, which correlates with symptomatic improvement, suggesting a multifaceted mechanism beyond monoaminergic systems [11].

Beyond ADHD and narcolepsy, MPH is being investigated for off-label use in stimulant use disorder, particularly cocaine dependence. The rationale stems from MPH’s ability to normalize dysregulated dopaminergic signaling and improve executive control deficits, thereby potentially reducing impulsivity and drug-seeking behaviors [12–14]. However, this therapeutic avenue is balanced by concerns over MPH’s abuse potential, which necessitates careful patient selection, risk assessment, and ongoing monitoring during treatment [12–14].

Safety considerations include cardiovascular effects such as tachycardia, hypertension, and, rarely, severe cardiac events, alongside psychiatric adverse effects like anxiety, irritability, and exacerbation of psychotic symptoms - particularly in vulnerable individuals (e.g., children with comorbidities, individuals with history of substance use, or pre-existing psychiatric disorders) [15, 16]. Longitudinal studies underscore the importance of vigilance regarding neuropsychiatric and cardiovascular risks, especially in long-term treatment contexts [17, 18]. Despite these concerns, current guidelines emphasize individualized treatment strategies integrating risk-benefit analyses to maximize clinical efficacy while minimizing harm [1–3, 19].

Despite its established efficacy, MPH safety profile remains an area of active clinical and regulatory scrutiny. Cardiovascular adverse effects are among the most consistently reported concerns, encompassing increased heart rate, elevated blood pressure, and, in rare instances, severe outcomes such as arrhythmias or sudden cardiac events [15, 16]. These effects are particularly relevant in individuals with preexisting cardiac conditions, necessitating pre-treatment cardiovascular assessment and routine monitoring during therapy [1–3, 15]. Psychiatric adverse events also merit attention; MPH has been associated with heightened anxiety, irritability, mood lability, and, in susceptible individuals, the exacerbation or emergence of psychotic symptoms [15, 16]. These neuropsychiatric risks may be especially pronounced in patients with comorbid mood or psychotic disorders, underlining the need for individualized treatment planning and vigilant clinical surveillance [17, 18].

Another critical concern pertains to the abuse liability of MPH, which is intrinsically linked to its dopaminergic mechanism of action. By inhibiting the dopamine transporter and increasing extracellular dopamine levels, particularly in striatal regions implicated in reward processing, MPH possesses reinforcing properties that may predispose to misuse or dependence, especially in non-medical contexts or in individuals with a history of substance use disorders [5, 12–14]. While structured treatment settings and extended-release formulations may mitigate this risk, cautious patient selection and comprehensive risk-benefit evaluation remain imperative [12–14].

According to a recent Cochrane review, the certainty of evidence supporting the efficacy of MPH and other stimulant medications for treating ADHD in children and adolescents is low. This is primarily due to limitations in study quality, inconsistency of results, and imprecision, highlighting the necessity for further high-quality research to more definitively assess the benefits and risks of these treatments in this population [20]. In adults, a recent network meta-analysis by Ostinelli et al. identified stimulants and atomoxetine as the only pharmacological interventions with demonstrated short-term efficacy in reducing core ADHD symptoms, as evidenced by both self-reported and clinician-reported measures. However, these medications did not show significant improvements in quality of life, and the evidence concerning their long-term effectiveness remains limited and insufficiently explored [21].

Given the widespread and often long-term use of MPH, particularly among children, adolescents, and adults with persistent ADHD, a thorough understanding of its comprehensive risk profile is essential to optimize clinical outcomes and ensure patient safety. Chronic administration raises concerns regarding cumulative adverse effects that may not be fully apparent in short-term trials, including potential impacts on cardiovascular function, neurodevelopment, and psychiatric comorbidities. Moreover, the heterogeneity of patient populations - encompassing varying ages, comorbid conditions, and genetic predispositions - necessitates individualized risk assessment and management strategies. This review synthesizes the most current and robust evidence regarding the adverse effects associated with MPH treatment, focusing specifically on cardiovascular, psychiatric, and dependency-related outcomes, which represent the domains of highest clinical relevance and regulatory scrutiny. Data from available preclinical studies, randomized controlled trials, observational cohorts, and meta-analyses are critically appraised to elucidate the nuanced balance between therapeutic efficacy and safety risks. Emphasizing the principles of precision medicine, ongoing patient monitoring, tailored dosing regimens, and integration of risk mitigation strategies are recommended to maximize clinical benefit while minimizing potential harms, thereby supporting informed decision-making in both pediatric and adult ADHD management [1–3, 17–19].

## Methodology

This manuscript was developed as a comprehensive narrative review synthesizing the current literature on pharmacological and non-pharmacological management strategies for ADHD, with a focused emphasis exclusively on methylphenidate (MPH). This deliberate focus was chosen to maintain conceptual clarity and avoid diluting the analysis by including other stimulant medications, despite their clinical relevance, as MPH remains one of the most widely prescribed and studied stimulants for ADHD treatment worldwide [22].

The methodology prioritized inclusion of recent, high-quality, peer-reviewed scientific publications, authoritative clinical guidelines, and regulatory documentation available up to 2025. Systematic searches were performed in major biomedical databases, including PubMed, Cochrane Library, and ClinicalKey, using keywords such as “methylphenidate,” “ADHD,” “pharmacotherapy,” “efficacy,” “safety,” “adverse effects,” “cardiovascular,” “psychiatric outcomes,” and “abuse potential.” The search targeted randomized controlled trials, meta-analyses, systematic reviews, and pharmacological studies evaluating MPH’s efficacy, safety, adverse events, and long-term outcomes. To ensure consistency and relevance, inclusion criteria were predefined as follows: studies published in English up to 2025, involving human subjects diagnosed with ADHD across all age groups (children, adolescents, adults), and focusing on methylphenidate’s efficacy, safety, or adverse effects. Both randomized controlled trials and observational studies were considered, with priority given to peer-reviewed articles and authoritative guidelines. Non-peer-reviewed literature and animal-only studies were excluded unless they provided essential mechanistic insights. The selection process involved initial screening based on titles and abstracts, followed by full-text review of potentially relevant articles. Two independent reviewers performed screening and resolved discrepancies by consensus to minimize selection bias. This approach, although not a formal systematic review, aimed to maintain rigor and transparency in source inclusion. To provide a comprehensive safety overview, regulatory drug labels, pharmacovigilance databases, and post-marketing surveillance reports were incorporated. Emphasis was placed on studies assessing neurological and psychiatric sequelae, cardiovascular safety, and abuse and dependence liability related to MPH use. This facilitated a multidimensional synthesis addressing MPH’s clinical effectiveness across diverse populations, pathophysiological mechanisms underlying ADHD, and treatment response, and pragmatic considerations in clinical decision-making. Special attention was given to contextualizing findings relative to age-specific treatment paradigms, comorbid psychiatric and somatic conditions, and differential therapeutic modalities, including behavioral and cognitive interventions. Identified knowledge gaps and areas requiring further empirical study were highlighted to guide future research. While the narrative review format precludes exhaustive systematic synthesis, it allows integration of diverse evidence types, including mechanistic studies, clinical trials, and real-world data, providing a comprehensive perspective on MPH use in ADHD. This methodology facilitates addressing complex clinical questions where heterogeneity and emerging data preclude strict meta-analytic approaches.

The ultimate aim of this narrative review is to provide a balanced, evidence-based, and clinically relevant overview of MPH use in ADHD management, supporting healthcare professionals in optimizing individualized treatment strategies that maximize therapeutic benefit while minimizing potential risks.

A synthesis of the most recent knowledge on minimizing the risks associated with MPH use can be framed within the context of current treatment guidelines. These guidelines have been expanded to address not only adults but also minors, reflecting the growing recognition of ADHD as a lifelong condition. Consequently, the continuation of treatment from childhood or adolescence into adulthood is increasingly common, necessitating careful and ongoing safety monitoring.

## Expert guidelines for the treatment of children, adolescents, and adults with ADHD, developed in accordance with institutional protocols

The National Institute for Health and Care Excellence (NICE), Canadian ADHD Resource Alliance (CADDRA), and Australian ADHD Professionals Association (AADPA) guidelines address ADHD treatment comprehensively, encompassing both non-pharmacological and pharmacological interventions. MPH is listed as one of several therapeutic options, alongside other medications such as lisdexamfetamine, atomoxetine, and guanfacine. Treatment selection should be individualized based on the patient’s specific needs, age, side effect profile, and response to previous therapies. Therefore, while MPH is an important option, it is one among several recommended pharmacotherapeutic agents for ADHD according to the NICE, CADDRA, and AADPA guidelines (detailed recommendations are provided in Tables 1 and 2). In the NICE guidelines 2018, updated in 2019 and 2020 (United Kingdom), a comprehensive approach is recommended for the treatment of ADHD, combining non-pharmacological therapy (psychological support, behavioral therapy, cognitive therapy, school assistance, support for caregivers), diet, and physical exercise – with pharmacological therapy. All treatment proposals should be discussed with the patient and caregivers (in the case of minors) [2].

General Risk Management Rules have been established for treatment to optimize therapy safety, minimize adverse effects, and ensure effective patient monitoring. These rules include thorough assessment of treatment indications and contraindications, regular monitoring of clinical and laboratory parameters, as well as educating patients and their caregivers on recognizing and reporting potential adverse events.

Children under 5 years of age should not be prescribed medication for ADHD. For children over 5 years old, pharmacological treatment should be considered only if non-pharmacological interventions fail to produce significant improvement. In adults, medication is recommended when non-pharmacological therapies do not yield adequate benefit. Prescription of medication must be carried out exclusively by qualified physicians, following confirmation of the ADHD diagnosis and thorough evaluation of the patient’s medical history and current health status, including the documented ineffectiveness of non-pharmacological approaches. After diagnosis, patients, their caregivers (for minors), and clinicians should collaboratively determine the most appropriate treatment strategies, including the sequence and timing of interventions to be initiated or trialed [19].

Individuals with ADHD should be actively involved in decisions regarding their own care, in accordance with their age and developmental level. Clinicians are responsible for providing comprehensive information about available treatment options, including their benefits and potential adverse effects. Additionally, the acceptability and feasibility of each treatment must be evaluated on an individual basis, taking into account factors such as age, geographic location, available resources, and healthcare service capacity [2].

The NICE evidence review found that pharmacological treatment was more effective than non-pharmacological treatment in reducing core ADHD symptoms. Combined pharmacological and non-pharmacological treatment was better than either alone. Each mode was more effective than the other in targeting specific aspects of ADHD: pharmacological treatments were more effective for reducing core ADHD symptoms, and non-pharmacological treatments were more effective for improving functional outcomes for people with ADHD [2]. There is currently no evidence from which to ascertain whether it is generally more effective to start treatment with pharmacological approaches or non-pharmacological approaches, or the optimal time to start treatment. In the absence of direct evidence, these decisions should consider availability, cost, preferences, and potential harms. Non-pharmacological treatments can be combined with medication. If medication is not effective enough, non-pharmacological treatments can be added to the treatment plan. Alternatively, if non-pharmacological approaches are tried first, and functional impairment remains, medication can be added [19].

A combined approach to treating ADHD is advantageous because pharmacological and non-pharmacological therapies target distinct aspects of the disorder. Current evidence supports pharmacological treatments as the most effective for reducing core ADHD symptoms, while non-pharmacological interventions may be more beneficial in enhancing functional outcomes in affected individuals. Importantly, treatment plans should also address frequently co-occurring conditions such as mood instability, anxiety, and depression, integrating management strategies for these comorbidities according to established guidelines for each specific disorder [19]. Combined treatment is recommended when it is accessible, feasible, and cost-effective within the local healthcare context, and when the selected approach effectively addresses the individual’s symptoms, functional impairments, or participation needs. This strategy is particularly advised for patients who demonstrate insufficient response to either pharmacological or non-pharmacological interventions alone [19]. These decisions should consider potential adverse effects and costs, both direct and indirect. Treatment effects should be monitored for effectiveness, including treatment-specific outcomes and adverse effects. Timing of the effect of intervention may also be a factor, given stimulant medication works immediately, whereas some non-stimulant medications may take several weeks to have an effect, and similarly for non-pharmacological treatments [23].

These recommendations are based on the current evidence, which indicates that combined treatments are more effective in treating ADHD symptoms than either pharmacological treatment or non-pharmacological treatment in isolation and that this benefit is larger and more consistently observed when compared with non-pharmacological treatment. Multimodal treatment allows for a tailored approach. The clinical profile may guide treatment decisions. For example, non-stimulant medications may be indicated for a person with co-occurring Tourette syndrome. The NICE committee concluded that there was insufficient evidence to make strong recommendations about any sequence or combinations of treatments. It is also important to note that ADHD medications will not provide full coverage over the course of a day/evening. Non-pharmacological therapy can assist with the development of strategies and skills to maximize functioning at such times [2].

**Table 1:** Comparative summary of Pharmacological treatment recommendations for ADHD in children and adolescents aged 5–17 years, based on three major clinical guidelines: AADPA (2022), NICE (2019), and CADDRA (2020)

Origins	1st line treatment	2nd line treatment	3rd line treatment
AADPA 2022 [19] Australia	MPH or Dexamphetamine or Lisdexamfetamine	Switch to another stimulant if the preparation is ineffective or poorly tolerated • Choosing a drug based on advantages and disadvantages or short and long duration of action, as well as patient/caregiver/family preferences	Atomoxetine, guanfacine or clonidine • in case of poor response, poor tolerance or contraindications to the use of stimulants or if adjunctive therapy is considered necessary
NICE 2019 [2] Great Britain	MPH (IR or ER)	Lisdexamfetamine or dexamfetamine* *if lisdexamfetamine is not well tolerated • After a 6-week trial with an appropriate dose, if there is insufficient reduction in ADHD symptoms and related disorders, you should move on to second-line treatment	Atomoxetine or guanfacine • After 6 weeks, move to third-line treatment if symptoms do not respond to treatment or if the patient is intolerant to methylphenidate or lisdexamfetamine
CADDRA 2020 [23] Canada	Long-acting stimulants	Atomoxetine, guanfacine, short/rapid acting stimulants • Before initiating second-line treatment, an adequate trial of long-acting stimulants from both classes should be performed.	Bupropion, clonidine, imipramine, modafinil, atypical antipsychotics • They can also be used as adjunctive therapy or when stimulants are contraindicated, poorly tolerated or associated with suboptimal response

Abbreviations: AADPA - Australian ADHD Professionals Association; NICE - National Institute for Health and Care Excellence; CADDRA - Canadian ADHD Resource Alliance, MPH - methylphenidate, IR - short release, ER- extended release; ADHD - Attention-Deficit Hyperactivity Disorder

**Table 2:** Comparative summary of Pharmacological treatment recommendations for ADHD in adults, based on three major clinical guidelines: AADPA (2022), NICE (2019), and CADDRA (2020)

Origins	1st line treatment	2nd line treatment	3rd line treatment
AADPA 2022 [19] Australia	MPH or Dexamphetamine or Lisdexamfetamine	Changing to another stimulant if the preparation is ineffective or poorly tolerated • Choosing a drug based on advantages and disadvantages or short and long duration of action, as well as patient/caregiver/family preferences	Atomoxetine, guanfacine or clonidine • in case of poor response, poor tolerance or contraindications to the use of stimulants or if supportive treatment
NICE 2019 [2] Great Britain	MPH or Lisdexamfetamine	Change to another stimulant if the drug is ineffective or poorly tolerated *dexamfetamine if lisdexamfetamine is not well tolerated • After a 6-week trial with an appropriate dose, if there is insufficient reduction in ADHD symptoms and related disorders, you should move on to second-line treatment	Atomoxetine • After 6 weeks, move to third-line treatment if symptoms do not respond to treatment or if the patient is intolerant to methylphenidate or lisdexamfetamine
CADDRA 2020 [23] Canada	Long-acting stimulants	Atomoxetine, guanfacine, short/rapid acting stimulants • Before initiating second-line treatment, an adequate trial of long-acting stimulants from both classes should be performed.	Bupropion, clonidine, imipramine, modafinil, atypical antipsychotics • They may also be used as adjunctive therapy or when stimulants are contraindicated, poorly tolerated or associated with a suboptimal response.

Abbreviations: AADPA - Australian ADHD Professionals Association; NICE - National Institute for Health and Care Excellence; CADDRA - Canadian ADHD Resource Alliance, MPH - methylphenidate, IR - short release, ER- extended release; ADHD - Attention-Deficit Hyperactivity Disorder

A healthcare professional with training and expertise in managing ADHD should review ADHD medication at least once a year and discuss with the person with ADHD (and their families and carers as appropriate) whether medication should be continued. Patients with ADHD should be encouraged to openly discuss their preferences regarding discontinuation or modification of medication and to actively participate in decisions related to stopping treatment. Trial periods of medication cessation or dose reduction should be considered when the overall assessment of benefits and risks indicates that such an approach may be appropriate. If the decision is to continue medication, the rationale for this choice must be thoroughly documented [2].

## Adverse effects of methylphenidate

### Cardiac adverse effects

MPH has been associated with modest increases in blood pressure, typically ranging from 2 to 4 mmHg, and heart rate elevations of approximately 3 to 6 beats per minute. These changes are generally considered clinically insignificant in most healthy children, adolescents, and adults [18, 24]. However, in individuals with preexisting cardiac conditions, the risk of serious adverse events, including sudden cardiac death, is significantly elevated [9]. An increased risk of arrhythmias is particularly notable within the first eight weeks of treatment, necessitating close cardiovascular monitoring, especially in patients with structural heart defects or a history of arrhythmias [8].

The potential cardiotoxic effects of MPH, such as tachycardia, hypertension, or valvulopathy, remain a matter of ongoing debate. In vitro studies have demonstrated that MPH binds to serotonin 5-HT2B receptors, whose activation has been implicated in the pathogenesis of valvular heart disease [9]. However, the precise pharmacological profile of this interaction, including whether MPH acts as an agonist or antagonist at the 5-HT2B receptor, requires further elucidation. Preliminary pharmacological evidence also suggests that MPH may function as an agonist at the 5-HT1A receptor [10], which warrants additional research into the mechanistic underpinnings and potential clinical implications of these receptor interactions. It seems that cardiovascular monitoring is essential, especially in patients with structural heart defects or a history of arrhythmias.

### Psychiatric adverse effects

MPH has been associated with the exacerbation of preexisting psychotic disorders, the induction of manic episodes, and the emergence of novel psychiatric symptoms, such as behavioral dysregulation and disturbances in thought content [25–27]. Consequently, a thorough assessment of both personal and familial psychiatric history is essential before the initiation of MPH therapy. Moreover, patients should be routinely monitored for new-onset or worsening psychiatric manifestations throughout treatment.

A comprehensive synthesis of available data on the neurological and psychiatric outcomes of long-term MPH administration in individuals with ADHD, as presented by Krinzinger et al. [28], highlights a complex risk-benefit profile. While some reports suggest an increased risk of psychotic symptoms and tic disorders during extended use, case studies indicate that such symptoms may resolve upon discontinuation of the drug. Conversely, there is also evidence indicating potential protective effects of MPH against depressive symptoms and suicidal behavior in individuals with ADHD.

Certain populations - such as preschool-aged children, patients with preexisting tic disorders, and adolescents vulnerable to substance use - may be at elevated risk for adverse neuropsychiatric effects and therefore warrant particular caution. Furthermore, the relationship between MPH-use and outcomes related to bipolar disorder or seizure threshold remains insufficiently understood due to the absence of high-quality, long-term data. The interplay between MPH, ADHD symptomatology, and sleep disturbances is also noted to be intricate, with many existing studies lacking clarity regarding long-term effects.

The cumulative evidence supports the use of MPH in ADHD, particularly given its potential for favorable neuropsychiatric outcomes. Nonetheless, long-term treatment should be managed under specialist supervision, with structured, ongoing evaluation. The authors underscore the importance of future research utilizing large-scale longitudinal datasets, with an emphasis on specific neuropsychiatric endpoints and comparative analyses between long-term pharmacotherapy and alternative or shorter-duration interventions [15].

Although MPH is widely used to improve attention and behavioral control in individuals with ADHD, concerns have been raised about potential adverse effects on affect, motivation, and creativity, particularly in adolescents. In a qualitative investigation by Kovshoff et al. (2019), both adolescents and caregivers reported instances of emotional blunting, reduced spontaneity, and diminished motivation during stimulant treatment, which in some cases led to medication discontinuation due to a perceived loss of authenticity or affective flatness [28]. Similarly, a review by Hoogman et al. (2020) summarizing behavioral and neurocognitive findings indicated that psychostimulants may reduce divergent thinking and creative ideation in some individuals, although results across studies are heterogeneous [29]. These findings underscore the importance of individualized monitoring, as stimulant treatment may, in a subset of patients, inadvertently suppress key dimensions of personality and cognitive flexibility.

However, researchers remain divided on the extent to which MPH affects higher-order cognitive functions such as creative thinking. In an earlier study by Baas et al. (2020), no significant effects of MPH were observed on either convergent or divergent creativity in healthy adults, suggesting that the drug may not impair creative processes in individuals without baseline cognitive deficits [30]. In contrast, a more recent investigation by Sayalı et al. (2023) demonstrated that the impact of MPH on divergent thinking is modulated by individual differences in baseline dopamine synthesis capacity. Using PET imaging, this study revealed that MPH reduced idea variability, a key indicator of divergent creativity in participants with low baseline dopamine synthesis, while enhancing it in those with higher dopaminergic capacity [31]. These findings suggest that the cognitive effects of MPH, particularly in domains such as creativity, may depend on neurochemical individual differences, thereby offering a potential explanation for the inconsistencies reported across the literature. For example, in a randomized, double-blind, placebo-controlled crossover study utilizing a simulated adult workplace environment (AWE), Wigal et al. (2010) demonstrated that lisdexamfetamine dimesylate significantly improved attention and executive functioning in adults with ADHD. Importantly, the study also reported a comprehensive safety profile, including adverse effects, making it a valuable reference point for comparative discussions on the efficacy and tolerability of stimulant medications such as MPH in adult populations [32].

A large register-based cohort study by Chen et al. (2014) investigated the association between pharmacological treatment for ADHD and suicidal behavior in over 37,000 individuals aged 12–25 years. The findings indicated that the risk of suicide-related events was significantly lower during periods of ADHD medication use, predominantly MPH and amphetamines, compared to periods without treatment. These results suggest that, contrary to some prior concerns, stimulant treatment may have a protective effect against suicidal behavior when appropriately prescribed and monitored [33].

### Risk of abuse and dependence

MPH is designated as a Schedule II controlled substance, reflecting its recognized potential for abuse and dependence [25, 34]. This classification necessitates stringent regulatory oversight and underscores the importance of patient education, particularly among adolescents and young adults, regarding the proper use, storage, and disposal of the medication. Regulatory guidance, including the boxed warning, stresses the need for vigilant prescribing practices and systematic monitoring to reduce the risk of misuse, diversion, and the development of substance use disorders [35].

In parallel with these considerations, a broader discussion has emerged around the potential role of controlled psychostimulants, including MPH, in the treatment of stimulant use disorder (StUD). In their analysis, Suen et al. [27] examine the legal and clinical implications of prescribing such agents in the context of rising mortality linked to the use of illicit stimulants, including amphetamines and cocaine. While preliminary studies suggest that pharmacotherapies such as dextroamphetamine, MPH, and modafinil may offer symptom reduction and lower stimulant consumption, current prescribing rates remain limited. This has raised questions about both the strength of existing evidence and its applicability to broader clinical populations. Although the authors advocate for expanded access to these pharmacological options, the absence of FDA-approved treatments for StUD warrants a cautious interpretation of the available data and calls for more rigorous, large-scale investigations to determine efficacy, safety, and real-world effectiveness before widespread adoption can be justified [27].

Notably, ADHD is a well-established risk factor for developing substance use disorders (SUD), with comorbidity rates significantly exceeding those in the general population. Neurocognitive mechanisms underlying this association include deficits in executive function, impulsivity, and dysregulation of motivational and reward-related brain circuits, particularly relevant in the context of increasing cannabis use among individuals with ADHD [36].

### Common adverse effects

The most commonly observed adverse effects associated with MPH include insomnia, appetite suppression, nausea, vomiting, abdominal discomfort, weight loss, and headaches [36, 37]. These reactions frequently exhibit a dose-dependent relationship and may necessitate titration or, in some cases, consideration of alternative pharmacological options. MPH exerts its anorexigenic effects through its amphetamine-like mechanism, notably influencing dopaminergic and noradrenergic pathways involved in appetite regulation.

Data from the UK Yellow Card Scheme identify anorexia as the most frequently reported adverse event among children receiving MPH for ADHD, accounting for approximately 34% of all reports in this population [16]. These findings are consistent with data from the WHO’s global pharmacovigilance database, VigiBase. Among children aged 2 to 11 years - both those receiving ADHD pharmacotherapy within the approved indication and others in the same age group - psychostimulants used for ADHD treatment contribute to 14% of adverse drug reaction (ADR) reports, with anorexia listed as the predominant symptom [38].

### Long-term safety in studied populations

Some long-term studies, including the Attention Deficit Hyperactivity Disorder Drugs Use Chronic Effects (ADDUCE) study, which followed participants for 24 months, suggest that MPH does not significantly impact growth, psychiatric stability, or neurological function in children and adolescents [7]. Persistent elevations in heart rate and blood pressure have been observed during MPH treatment, underscoring the importance of routine cardiovascular assessment in pediatric populations. Notably, the ADDUCE study reported a measurable decline in weight gain velocity at the 6-month follow-up, a finding that aligns with prior evidence implicating MPH’s anorexigenic effects as a predominant adverse reaction in children with ADHD [7]. Reduced appetite and associated impairments in growth trajectories represent clinically significant risks that warrant vigilant long-term monitoring.

In terms of neuropsychiatric outcomes, the study documented a less pronounced reduction in tic severity among MPH-treated individuals relative to untreated controls. Furthermore, although the prevalence of suicidal ideation and behavior appeared numerically lower in the non-MPH group, statistical significance was not achieved. Specifically, the odds ratio for reduced suicidality favored the non-treated group (OR = 2.28, 95% CI: 0.64–8.11), yet the limited sample size (MPH: *n* = 756; no MPH: *n* = 391) likely reduced the study’s power to detect significant differences for rare events. These findings exemplify the challenge of Type II error in underpowered longitudinal studies and should therefore be interpreted with caution [7].

Despite being on the market for almost 60 years, the tolerance of MPH has never been studied in detail. One study from the UK Medicines and Healthcare Products Regulatory Agency, which collects suspected drug reactions in patients aged < 17 years, reported that, excluding vaccines, MPH was the medication most commonly associated with adverse reactions in children [16].

The long-term safety of MPH use is poorly documented. For example, the long-term implications of continuous treatment from childhood to adulthood in terms of height or long-term changes in the brain are unknown. The Cochrane review of Cândido et al. included 10 clinical trials with 497 adult participants, primarily outpatients, with treatment durations ranging from 6 to 18 weeks. Six trials compared IR MPH to placebo, while the remaining studies compared it to bupropion, lithium, osmotic-release oral system (OROS) MPH, and Pycnogenol^®^. One trial with 146 participants suggested that IR MPH might reduce ADHD symptoms, showing a moderate positive effect when participants assessed their own symptoms. However, these results were uncertain and could change with additional data. IR MPH had little to no effect on anxiety and depression symptoms. When compared to lithium, IR MPH did not significantly impact ADHD symptoms or mental health outcomes, though results remained uncertain. Risk assessment revealed multiple concerns regarding the study methodologies and potential conflicts of interest, which may have affected the reliability of the findings. Adverse events were poorly assessed and reported, but four trials involving 203 participants receiving IR MPH and 141 receiving placebo reported an increased risk of gastrointestinal issues and appetite loss. Heart-related adverse events were reported sporadically and inconsistently. Overall, most trials raised significant concerns regarding funding sources and conflicts of interest, further undermining the credibility of the results [39].

In a comprehensive analysis of pediatric adverse drug reaction (ADR) reports submitted to the UK Yellow Card Scheme between 2000 and 2009, Hawcutt et al. (2012) identified stimulant medications, including MPH, among the drug classes most frequently associated with neuropsychiatric ADRs in children. The study emphasizes the challenges of establishing causality in spontaneous reporting systems and the potential overrepresentation of chronically used medications. These findings highlight the importance of cautious interpretation of post-marketing surveillance data and support the integration of such data with other pharmacovigilance sources when assessing the safety profile of MPH [40].

### Data from clinical trials and neurochemical effects studies

Magnetic resonance spectroscopy (MRS) has been employed to investigate the neurochemical underpinnings of MPH treatment in adults with ADHD. A notable double-blind, placebo-controlled study assessing the effects of 12-week MPH administration revealed no significant changes in glutamate or glutamate-plus-glutamine (Glx) concentrations within the anterior cingulate cortex and cerebellum despite clinical improvement in core ADHD symptoms [11]. These findings suggest that short-term MPH treatment may not modulate glutamatergic neurotransmission at the macroscopic level detectable by MRS, indicating that the therapeutic efficacy of MPH might involve other neurochemical or circuit-level mechanisms [11]. This study’s limited sample size and treatment duration constrain the extrapolation of results to long-term neurochemical adaptations. Authors concluded that there is a need for future longitudinal research involving larger participant groups and prolonged treatment durations to better characterize the long-term neurochemical effects of MPH and how these relate to clinical outcomes. Such studies may help determine whether continuous MPH administration promotes adaptive neuroplastic changes that underlie enduring symptom reduction and functional gains in adults with ADHD [11]. One randomized, double-blind, placebo-controlled trial investigated the age-dependent effects of MPH on the dopaminergic system in male patients with ADHD. The study included stimulant-naïve children aged 10–12 years and adults aged 23–40 years. Findings indicated that 16 weeks of MPH treatment increased the cerebral blood flow response to a dopamine challenge in the thalamus and striatum of children but not in adults, suggesting age-related differences in MPH’s impact on the dopaminergic system. Additionally, differences in striatal dopamine release patterns between the two age groups implied that age-related neurodevelopmental changes influence MPH’s pharmacodynamics. However, the study was limited by its cross-sectional design, which prevents the assessment of long-term treatment outcomes, and its reliance on imaging biomarkers without direct clinical outcome measures [41].

A 6-year prospective naturalistic study investigated the long-term tolerability and safety of pharmacological treatment in adults with ADHD. Among 112 participants, 57 (51%) continued MPH treatment at follow-up, while 55 (49%) had discontinued. The main reasons for discontinuation were lack of efficacy (29%), elevated mood or hypomania (11%), and loss of contact with the prescribing physician (9%). Common adverse effects reported by those still on MPH included decreased appetite (28%), dry mouth (24%), anxiety/restlessness and increased pulse rate (19% each), decreased sexual desire (17%), and sweating (15%). Patients continuing treatment reported improved quality of life, higher functioning levels, and better self-awareness compared to nonmedicated individuals. The data showed no evidence of tolerance development or dosage escalation over time, a concern often discussed in the literature. Long-term medication use might facilitate benefiting from nonpharmacological interventions, potentially allowing dose reduction. Limitations of the study include the lack of psychiatric comorbidity assessment at follow-up, although baseline comorbidity rates were similar between those who continued and discontinued treatment. Additionally, changes in nonpharmacological or supportive interventions since baseline may have biased results. The study’s naturalistic, descriptive design and limited sample size (without power analysis) further restrict generalizability [18]. Findings are consistent with other published data [24].

A clinical trial compared the efficacy of MPH extended-release capsules with OROS-MPH, demonstrating differences in onset and duration of effect. The extended-release capsules provided a more gradual onset and a smoother therapeutic effect over 12 h, whereas OROS-MPH exhibited a more rapid onset but a sharper decline in efficacy towards the end of the dosing interval. These findings emphasize the need for individualized treatment selection based on patient-specific needs and symptom control patterns. However, the study lacked long-term follow-up data, leaving uncertainty about sustained efficacy and safety over extended treatment periods [42].

A 4-week double-blind, placebo-controlled trial established the efficacy of dex-MPH, the active d-isomer of MPH, in subjects aged 6 to 17 years. The study found that dex-MPH led to greater symptom reduction compared to racemic MPH while also presenting a lower incidence of adverse effects such as appetite suppression and insomnia. These results support the potential benefits of using the purified d-isomer formulation in pediatric ADHD treatment. However, the short trial duration limits understanding of long-term safety and efficacy, particularly regarding growth effects and psychiatric outcomes in children [43].

Data from the French Pharmacovigilance Database (1985–2011) reveal significant off-label use and associated adverse drug reactions (ADRs) related to MPH. In children under six years, MPH was mainly prescribed for ADHD, autism spectrum disorder, and psychomotor disorders. Among patients aged 6 to 18 years, off-label indications included behavioral disorders (38%), instability (19%), autism (13%), narcolepsy (13%), and intellectual disability (8.8%). In adults, 84% of reported MPH prescriptions were off-label, primarily for ADHD (13%), narcolepsy/hypersomnia (13%), depression (5%), and Parkinson’s disease (5%). A total of 181 ADRs were reported, involving 143 children (mean age 10 years), with 32% of pediatric cases representing off-label use. Serious ADRs accounted for 31.3% of on-label and 43% of off-label pediatric reports. In adults, serious ADRs constituted 33% of on-label and 52% of off-label cases. Neuropsychiatric effects were the most common ADRs, followed by cardiovascular and cutaneous events (13.3% each). Muscular effects (7.2%), misuse (5.5%), and less frequent complications such as lesions and intoxications (4.4%) were also reported. The incidence of spontaneous ADR reports increased steadily, with off-label use comprising approximately half of all notifications by 2011. Despite strict MPH prescribing regulations in France, over 40% of reported ADRs involved off-label use, which was associated with a higher frequency of serious events. These findings highlight the importance of cautious MPH prescribing and careful monitoring, especially in off-label contexts [3].

### Classification of adverse effects: Summary and recommendations

Adverse effects of MPH can be broadly classified into four main categories. First, there are acute adverse effects that are typically transient and may subside with appropriate dose adjustments. Second, acute cardiovascular adverse effects, which in some cases can lead to serious complications such as hypertension, tachycardia, or arrhythmias. Third, acute neuropsychiatric adverse effects, including exacerbation of preexisting psychiatric conditions or emergence of new symptoms such as mood disturbances or psychosis. Lastly, chronic unwanted effects that may have long-term negative implications, potentially mediated by mechanisms affecting sleep regulation, appetite control, and cardiovascular function. This classification underscores the importance of careful monitoring and individualized risk-benefit assessment during MPH treatment.

Table 3 provides a summary of these categories with a description of specific adverse effects and recommendations. The first three categories are relatively well-documented. The last category is least understood due to the lack of longitudinal studies monitoring the development of patients treated with MPH from childhood to adulthood. At the same time, from a public health perspective, these are vital since they may translate into health risks at the level of the whole treated cohort of young patients. Therefore, systematic long-term studies monitoring the development of children and adolescents treated with MPH are warranted.

**Table 3:** Classification of methylphenidate-associated adverse effects by temporal profile and clinical domain, with recommended monitoring and management strategies

Category	Adverse effects	Recommendations
Acute - may subside after dose adjustments	• Insomnia • Decreased appetite • Nausea • Vomiting • Abdominal pain • Weight loss • Headache	• Careful observation of adverse effects • Dose adjustments or a change of treatment
Acute – serious adverse cardiovascular events	• Myocardial infarction • Arrhythmias • Sudden cardiac death	• Careful screening for cardiovascular risk • Monitoring of cardiovascular health, especially in patients with structural heart defects or a history of arrhythmias
Acute - may produce adverse neuropsychiatric complications	• Psychiatric adverse effects, including anxiety, agitation, psychotic symptoms • Exacerbation of preexisting psychotic or mood disorders • Inducing manic episodes • New-onset psychiatric symptoms, including behavior disturbances and thought disorders	• Careful screening for personal and familial psychiatric history • Monitoring for emergent psychiatric symptoms during treatment
Chronic – may contribute to long-term negative consequences via plausible mechanisms associated with sleep, appetite, and cardiovascular health	• Insomnia or decreased sleep time • Decreased appetite, weight loss, and slower trajectory of weight gain in children and adolescents • Increased heart rate and elevated blood pressure	• Careful observation of adverse effects • Monitoring of health • Monitoring of development in children and adolescents

## Conclusions

MPH remains one of the most effective pharmacological treatments for ADHD and narcolepsy, with emerging potential in conditions such as stimulant use disorder. However, its clinical use requires comprehensive patient assessment and continuous monitoring due to risks associated with cardiovascular and psychiatric adverse effects, as well as its potential for misuse and dependence. While current evidence suggests no major long-term adverse impact on growth or neurodevelopment in children, data on chronic exposure in adult populations are still limited.

The interindividual variability in treatment response underscores the need for personalized approaches, taking into account genetic, developmental, environmental, and psychosocial factors. Special attention should be given to commonly reported adverse effects such as appetite suppression and sleep disturbances, particularly in pediatric populations. Furthermore, comprehensive patient and caregiver education, along with regular health monitoring, are essential components of safe prescribing practice.

From a broader perspective, MPH therapy should ideally be embedded within a multimodal treatment framework, integrating behavioral interventions and psychoeducation. Advances in digital tools and biomarkers may support more refined treatment selection and monitoring in the future. Overall, prescribing MPH must remain a carefully balanced decision, informed by a nuanced evaluation of the benefit-to-risk ratio for each patient.

## Future directions

Future research should prioritize long-term, prospective, naturalistic studies that assess the safety and effectiveness of MPH in diverse and representative clinical populations. This is particularly important in adult patients, as the available high-quality data remains limited. Specific research attention is needed to evaluate the neurodevelopmental, cardiovascular, and metabolic consequences of prolonged exposure to MPH, with a focus on potential effects related to dose and treatment duration.

In addition, further investigation is warranted into the clinical implications of off-label prescribing and the co-administration of multiple psychotropic medications. These practices are becoming increasingly prevalent in the treatment of AHDH in adults and may influence both therapeutic outcomes and the overall safety profile of methylphenidate. High-quality longitudinal studies that follow individuals from childhood into adulthood are also essential for understanding how early exposure to this medication may influence developmental pathways over time.

In our opinion, closing these knowledge gaps will require not only well-designed clinical trials but also the implementation of integrated care models. Such models should combine pharmacological treatment with behavioral interventions and psychosocial support to optimize patient outcomes. Finally, regular updates to clinical practice guidelines are essential to ensure that prescribing remains evidence-based, clinically appropriate, and ethically responsible.

## Data Availability

No datasets were generated or analysed during the current study.

## Abbreviations

5–HT: 5–Hydroxytryptamine (Serotonin)
5–HT1A: 5–Hydroxytryptamine Receptor1A
5–HT2B: 5–Hydroxytryptamine Receptor 2B
AADPA: Australian ADHD Professionals Association
ADDUCE: Attention Deficit Hyperactivity Disorder Drug Use Chronic Effects Study
ADHD: Attention–Deficit Hyperactivity Disorder
ADR: Adverse drug reaction
bpm: Beats per minute
CADDRA: Canadian ADHD Resource Alliance
CBT: Cognitive–behavioral therapy
Cochrane: Cochrane database of systematic reviews
Cryo–EM: Cryo–electron microscopy
DAT: Dopamine transporter
ER: Extended release
FDA: Food and Drug Administration
hDAT: Human Dopamine Transporter
IR: Immediate release
mmHg: Millimeters of mercury
MPH: Methylphenidate
MR: Magnetic resonance
MRS: Magnetic resonance spectroscopy
NICE: National Institute for Health and Care Excellence
OR: Odds Ratio
OROS: Osmotic release oral system
SERT: Serotonin transporter
StUD: Stimulant use disorder
SUD: Substance use disorder
WHO: World Health Organization

## Declarations

### Bioethics

No human participants.

## References

[1] Kolar D, Keller A, Golfinopoulos M, et al. Treatment of adults with attention-deficit/hyperactivity disorder. Neuropsychiatr Dis Treat. 2008;4(2):389–403. 10.2147/ndt.s6985. PMID: 18728745 PMCID: PMC2518387. 18728745 10.2147/ndt.s6985 PMC2518387

[2] National Institute for Health and Care Excellence (NICE). Attention deficit hyperactivity disorder: diagnosis and management. NICE guideline NG87. London: NICE; 2018. [updated 2019]. https://www.nice.org.uk/guidance/ng87/chapter/Update-information. 29634174

[3] Trenque T, Herlem E, Taam MA, Drame M. Methylphenidate off-label use and safety. Springerplus. 2014;3:286. 10.1186/2193-1801-3-286. PMID: 25279275 PMCID: PMC4162523. 25279275 10.1186/2193-1801-3-286 PMC4162523

[4] Childress A, Tran C. Current investigational drugs for the treatment of attention-deficit/hyperactivity disorder. Expert Opin Investig Drugs. 2016;25(4):463–74. 10.1517/13543784.2016.1147558. PMID: 26814173. 26814173 10.1517/13543784.2016.1147558

[5] Li Y, Wang X, Meng Y, et al. Dopamine reuptake and inhibitory mechanisms in human dopamine transporter. Nature. 2024;632(8012):686–94. 10.1038/s41586-024-07796-0. PMID: 39112701. 39112701 10.1038/s41586-024-07796-0

[6] Kimko HC, Cross JT, Abernethy DR. Pharmacokinetics and clinical effectiveness of methylphenidate. Clin Pharmacokinet. 1999;37(6):457–70. 10.2165/00003088-199937060-00002. PMID: 10628897. 10628897 10.2165/00003088-199937060-00002

[7] Childress AC, Berry SA. The single-dose pharmacokinetics of NWP06, a novel extended-release methylphenidate oral suspension. Postgrad Med. 2010;122(5):35–41. 10.3810/pgm.2010.09.2199. PMID: 20861586. 20861586 10.3810/pgm.2010.09.2199

[8] Perrie Y, Rades T. Pharmaceutics: drug delivery and targeting. 2nd ed. London: Pharmaceutical; 2012.

[9] Andrejak M, Tribouilloy C. Drug-induced valvular heart disease: an update. Arch Cardiovasc Dis. 2013;106:333–9. 10.1016/j.acvd.2013.02.003. PMID: 23769407. 23769407 10.1016/j.acvd.2013.02.003

[10] Markowitz JS, DeVane CL, Ramamoorthy S, Zhu HJ. The psychostimulant d-threo-(R,R)-methylphenidate binds as an agonist to the 5HT1A receptor. Pharmazie. 2009;64:123–5. PMID: 19322953. 19322953

[11] Maier S, van Tebartz L, Philipsen A, et al. Effects of 12-week methylphenidate treatment on neurometabolism in adult patients with ADHD: the first double-blind placebo-controlled MR spectroscopy study. J Clin Med. 2020;9(8):2601. 10.3390/jcm9082601. PMID: 32796630 PMCID: PMC7464267. 32796630 10.3390/jcm9082601 PMC7464267

[12] Dürsteler KM, Berger EM, Strasser J, et al. Clinical potential of methylphenidate in the treatment of cocaine addiction: a review of the current evidence. Subst Abuse Rehabil. 2015;6:61–74. 10.2147/SAR.S50807. PMID: 26124696 PMCID: PMC4476488. 26124696 10.2147/SAR.S50807 PMC4476488

[13] Tardelli VS, Bisaga A, Arcadepani FB et al. Prescription psychostimulants for the treatment of stimulant use disorder: a systematic review and meta-analysis. Psychopharmacology (Berl). 2020;237(8):2233–2255. 10.1007/s00213-020-05563-3. PMID: 32601988 10.1007/s00213-020-05563-3 32601988

[14] Moeller SJ, Honorio J, Tomasi D, et al. Methylphenidate enhances executive function and optimizes prefrontal function in both health and cocaine addiction. Cereb Cortex. 2014;24:643–53. 10.1093/cercor/bhs345. PMID: 23162047 PMCID: PMC3920764. 23162047 10.1093/cercor/bhs345 PMC3920764

[15] Krinzinger H, et al. Neurological and psychiatric adverse effects of long-term methylphenidate treatment in ADHD: a map of the current evidence. Neurosci Biobehav Rev. 2019;107:945–68. 10.1016/j.neubiorev.2019.09.023. PMID: 31545988. 31545988 10.1016/j.neubiorev.2019.09.023

[16] Tobaiqy M, Stewart D, Helms PJ, et al. Parental reporting of adverse drug reactions associated with atenntion-deficit hyperactivity disorder (ADHD) medications in children attending specialist paediatric clinics in the UK. Drug Saf. 2011;34:211–9. 10.2165/11586050-000000000-00000. PMID: 21332245. 21332245 10.2165/11586050-000000000-00000

[17] Man KKC, Häge A, Banaschewski T, et al. Long-term safety of methylphenidate in children and adolescents with ADHD: 2-year outcomes of the ADDUCE study. Lancet Psychiatry. 2023;10(5):323–33. 10.1016/S2215-0366(23)00042-1. PMID: 36958362. 36958362 10.1016/S2215-0366(23)00042-1

[18] Edvinsson D, Ekselius L. Long-term tolerability and safety of Pharmacological treatment of adult ADHD. J Clin Psychopharmacol. 2018;38(4):370–5. PMCID: PMC6039396 PMID: 29927781 doi: 10.1097/JCP.0000000000000917. 29927781 10.1097/JCP.0000000000000917 PMC6039396

[19] Australian ADHD, Professionals Association. ADHD clinical guidelines. Melbourne: AADPA; 2022.

[20] Liguori S. What are the benefits and harms of methylphenidate in children and adolescents with attention-deficit/hyperactivity disorder? A Cochrane review summary with commentary. Dev Med Child Neurol. 2024;66(7):825–7. 10.1111/dmcn.15941. PMID: 38632839. 38632839 10.1111/dmcn.15941

[21] Ostinelli EG, Schulze M, Zangani C, Farhat LC, Tomlinson A, Del Giovane C, et al. Comparative efficacy and acceptability of pharmacological, psychological, and neurostimulatory interventions for ADHD in adults: a systematic review and component network meta-analysis. Lancet Psychiatry. 2025;12(1):32–43. 10.1016/S2215-0366(24)00360-2. PMID: 39701638 DOI:. 39701638 10.1016/S2215-0366(24)00360-2

[22] Lakhan SE, Kirchgessner A. Prescription stimulants in individuals with and without attention deficit hyperactivity disorder: misuse, cognitive impact, and adverse effects. Brain Behav. 2012;2(5):661–77. 10.1002/brb3.78. PMCID: PMC3489818 PMID: 23139911. 23139911 10.1002/brb3.78 PMC3489818

[23] Canadian ADHD, Resource Alliance (CADDRA). Canadian ADHD practice guidelines. 41 ed. ed. Toronto: CADDRA; 2020.

[24] Shellenberg TP, Stoops WW, Lile JA, Rush CR. An update on the clinical Pharmacology of methylphenidate: therapeutic efficacy, abuse potential and future considerations. Expert Rev Clin Pharmacol. 2020;13(8):825–33. 10.1080/17512433.2020.1796636. PMID: 32715789. 32715789 10.1080/17512433.2020.1796636

[25] Methylin. Label via DailyMed. Food and Drug Administration. Updated 2023-11-01.

[26] Ritalin. Label via DailyMed. Food and Drug Administration. Updated 2025-02-05.

[27] Suen LW, Coffin PO, Boulton KE, Carr DH, Davis CS. Prescribing psychostimulants for the treatment of stimulant use disorder: navigating the federal legal landscape. Am J Drug Alcohol Abuse. 2025;10(1097):1–6. 10.1097/ADM.0000000000001437. PMID: 39846466. 10.1097/ADM.0000000000001437 PMC13274479 39846466

[28] Kovshoff H, Banaschewski T, Buitelaar JK, Carucci S, Coghill D, Danckaerts M, et al. Reports of perceived adverse events of stimulant medication on cognition, motivation, and mood: qualitative investigation and the generation of items for the medication and cognition rating scale. J Child Adolesc Psychopharmacol. 2016;26(6):537–47. 10.1089/cap.2015.0218. PMID: 27007169 PMCID: PMC4991592. 27007169 10.1089/cap.2015.0218 PMC4991592

[29] Hoogman M, Stolte M, Baas M, Kroesbergen E, Creativity. A review of behavioral studies, the effect of psychostimulants and neural underpinnings. Neurosci Biobehav Rev. 2020;119:66–85. 10.1016/j.neubiorev.2020.09.029. PMID: 33035524. 33035524 10.1016/j.neubiorev.2020.09.029

[30] Baas M, Boot N, van Gaal S, de Dreu CKW, Cools R. Methylphenidate does not affect convergent and divergent creative processes in healthy adults. *Neuroimage*. 2020;15;205:116279. PMID: 31629831 10.1016/j.neuroimage.2019.116279 10.1016/j.neuroimage.2019.116279 31629831

[31] Sayalı C, van den Bosch R, Määttä JI, Hofmans L, Papadopetraki D, Booij J, Verkes RJ, Baas M, Cools R. Methylphenidate undermines or enhances divergent creativity depending on baseline dopamine synthesis capacity. Neuropsychopharmacology. 2023;48(13):1849–58. 10.1038/s41386-023-01615-2. PMID: 37270619 PMCID: PMC. 37270619 10.1038/s41386-023-01615-2 PMC10584959

[32] Wigal SB, Gao J, Chuang S, Paul J, Once-Daily Vyvanse Adult ADHD Study Group. Randomized, double‐blind, placebo‐controlled, crossover study of the efficacy and safety of Lisdexamfetamine dimesylate in adults with ADHD. Behav Brain Funct. 2010;6(34):1–14. 10.1186/1744-9081-6-34. PMID: 20576091 PMCID: PMC2908054. 20576091 10.1186/1744-9081-6-34 PMC2908054

[33] Chen Q, Sjölander A, Runeson B, D’Onofrio BM, Långström N, Lichtenstein P. Drug treatment for attention-deficit hyperactivity disorder and suicidal behaviour: register based study. BMJ. 2014;348:g3769. 10.1136/bmj.g3769. 24942388 10.1136/bmj.g3769 PMC4062356

[34] METADATE CD. Label via DailyMed. Food and Drug Administration. Updated 2023-10-31.

[35] Methylphenidate. Elsevier ClinicalKey. Drug Monograph. Content last updated: 2025-02-13.

[36] Gujska JH, Silczuk A, Madejek R, Szulc A. Exploring the link between Attention-Deficit hyperactivity disorder and Cannabis use disorders: A review. Med Sci Monit. 2023;29:e939749. 10.12659/MSM.939749. PMID: 37147797 PMCID: PMC10171029. 37147797 10.12659/MSM.939749 PMC10171029

[37] Methylphenidate-Class Stimulants for ADHD. Elsevier ClinicalKey. Drug Class Overview. Content last updated: 2023-11-04.

[38] Star K, Noren GN, Nordin K, Edwards IR. Suspected adverse drug reactions reported for children worldwide: an exploratory study using vigibase. Drug Saf. 2011;34:415–28. 10.2165/11587540-000000000-00000. PMID: 21513364. 21513364 10.2165/11587540-000000000-00000

[39] Cândido RCF, Menezes de Padua CA, Golder S, Junqueira DR. Immediate-release methylphenidate for attention deficit hyperactivity disorder (ADHD) in adults. Cochrane Database Syst Rev. 2021;1(1):CD013011. 10.1002/14651858.CD013011.pub2. PMID: 33460048; PMCID: PMC8092481. 33460048 10.1002/14651858.CD013011.pub2 PMC8092481

[40] Hawcutt DB, Mainie P, Riordan A, Smyth RL, Pirmohamed M. Reported paediatric adverse drug reactions in the UK 2000–2009. Br J Clin Pharmacol. 2012;73(3):437–46. 10.1111/j.1365-2125.2011.04113.x. PMCID: PMC3370348 PMID: 21988288. 21988288 10.1111/j.1365-2125.2011.04113.x PMC3370348

[41] Schrantee A, Tamminga HG, Bouziane C, Bottelier MA, Bron EE, Mutsaerts HJ. Age-Dependent effects of methylphenidate on the human dopaminergic system in young vs adult patients with Attention-Deficit/Hyperactivity disorder: A randomized clinical trial. JAMA Psychiatr. 2016;73(9):955–62. 10.1001/jamapsychiatry.2016.1572. PMID: 27487479 PMCID: PMC5267166. 10.1001/jamapsychiatry.2016.1572 PMC5267166 27487479

[42] Anbarasan D, Safyer G, Adler LA. Updates in Pharmacologic strategies in adult Attention-Deficit/Hyperactivity disorder. Child Adolesc Psychiatr Clin N Am. 2022;31(3):553–68. 10.1016/j.chc.2022.03.008. PMID: 35697401. 35697401 10.1016/j.chc.2022.03.008

[43] Childress AC. Stimulants. Child Adolesc Psychiatr Clin N Am. 2022;31(3):373–92. PMID: 35697391 10.1016/j.chc.2022.03.001. PMID: 35697391 10.1016/j.chc.2022.03.001 35697391

